# Convergence of obesity and high glycemic diet on compounding diabetes and cardiovascular risks in modernizing China: An emerging public health dilemma

**DOI:** 10.1186/1744-8603-4-4

**Published:** 2008-02-26

**Authors:** Eric L Ding, Vasanti S Malik

**Affiliations:** 1 Department of Nutrition, Harvard School of Public Health, Boston, Massachusetts, USA

## Abstract

As China is undergoing dramatic development, it is also experiencing major societal changes, including an emerging obesity epidemic, with the prevalence of overweight and obesity doubling in the past decade. However, the implications of a high glycemic index (GI) and glycemic load (GL) traditional Chinese diet are adversely changing in modern times, as a high-glycemic diet is becoming a greater contributor to diabetes and cardiovascular risks in a population with rising obesity and decreasing physical activity. Specifically, a high GI diet adversely impacts metabolism and appetite control regulation, and notably confers substantially greater risk of weight gain, type 2 diabetes, cardiovascular disease, and certain cancers among overweight and obese individuals (P<0.05 for all); leading to an emerging vicious cycle of compounding adverse health risks. Notably, while no elevated risk of cardiovascular disease and type 2 diabetes were observed with higher GL intake among normal weight individuals, among overweight individuals, higher GL was strongly associated with higher risk of coronary heart disease (RR=2.00, 95%CI: 1.31-2.96), stroke (RR=2.13, 1.28-3.53), and type 2 diabetes (RR=1.52, 1.22-1.89 among Chinese). Additionally, the influx of Western-diets rich in saturated fats and high-glycemic sugar-sweetened beverages also threaten the health of the population. This review highlights the emerging adverse convergence of a high-glycemic Asian diet with a Chinese society experiencing an emerging obesity epidemic, and the important implications of these combined factors on compounding cardiometabolic risks. Potential policy directions in China are also discussed.

## Introduction

Cardiovascular disease, diabetes, and cancer are not only leading causes of death in Western society, but have also recently become leading contributors of overall mortality in the People's Republic of China [1,2], where this is also a recent obesity epidemic [3-5]. From a nationally representative study, it is estimated that a large proportion of chronic disease mortality in China is attributable to physical inactivity, obesity, and obesity-related metabolic conditions [2]. Further exacerbating this problem is the convergence of a modernizing China and increasing obesity with a traditional high-glycemic Chinese diet, which together acts in tandem in increasing the risk of metabolic and cardiovascular diseases.

Lifestyle factors, such as nutrition, are recognized to play important roles in metabolic conditions such as obesity, diabetes, hypercholesterolemia, and cardiovascular diseases [6-15], as well as risk of cancer [11,16-22]. Recently, the quality of dietary carbohydrates has gained wide recognition as an important risk factor for disease. Whole-grain carbohydrates are regarded as more favorable while refined carbohydrates are regarded as more adverse for cardiovascular risk [9,15,23], not only due to their cereal fiber content but also for their glycemic index properties. The glycemic index (GI) and glycemic load (GL = GI * grams of carbohydrate) reflect the nature of carbohydrates in causing rapid postprandial increase in blood glucose and insulin levels [24,25], which have been rather consistently recognized to contribute to adversely impact a variety of metabolic risk factors. Notably a high GI/GL diet has been positively associated in multiple studies with weight gain and obesity in both animals [26] and humans [25,27,28], as well as higher levels of serum triglycerides, LDL cholesterol levels, and serum coagulation factors [25,29]. Increased insulin, as result of a high GI/GL diet, may also stimulate ovarian secretion of androgens, which has adverse metabolic consequences on risk of type 2 diabetes in women [30,31]. All thes mechanisms consequently leads to a high GI/GL diet being repeatedly shown to adversely effect glycemic control in individuals with diabetes [32], as well as associated with greater risk of developing type 2 diabetes [33-36], coronary heart disease [9,37-39] and stroke [40,41] in prospective studies.

Thus, an important risk factor relevant to Chinese society is the quality of dietary carbohydrate consumed as traditionally the Chinese diet consists of a variety of high-glycemic rice products as the staple grain, contributing as the primary source of caloric intake. While a high-glycemic Chinese diet did not formerly contribute to disease in an active and lean population, such a diet has important implications in a modernizing Chinese society characterized by increasing rates of adiposity, due an inherent biologic interaction in which high GI diet elicits significantly greater adverse effects in an overweight and obese population. This review highlights the emerging adverse convergence of a high-GI Asian diet with a Chinese society experiencing an emerging obesity epidemic, and the important implications of these combined factors on a series of compounding cardiometabolic risks and obesity-dependent conditions.

## Discussion

### Obesity in China

Like the rest of the world, China is experiencing an increased epidemic of obesity [3-5]. An estimated one-quarter of the Chinese population is overweight or obese [5,42]. A national survey in 2002 found that the prevalence of Chinese adult overweight and obesity has nearly doubled in the last 10 years [3] to 23% [5], with another national study estimating the prevalence at 27–31% in Chinese adults [43]. More dramatically, childhood overweight and obesity has substantially increased in China [3,44], from 1–2% in 1985 to 7–13% prevalence in larger Chinese cities in 2002 [3]. Additionally, in 2000 a study of adolescent students in 6 large Chinese cities found that the prevalence of childhood overweight and obesity has dramatically increased to over 14% for girls and 25% for boys [45]. A recent nationally-representative study in China comparing population obesity between 1990–1991 to 1999–2000 indicates that prevalence of overweight and obesity has substantially increased in all age groups and in all rural and urban areas across China, with obesity prevalence increasing by >2-fold in women to >3-fold in men [46].

When interpreting these findings it is important to consider that the adverse health ramifications of increasing rates of overweight and obesity may be of greater concern in Chinese than Western populations since it is now widely recognized that strong ethnic differences of how adiposity relates to glucose levels and cardiovascular risk factors exist [47-49]. For measures of adiposity such as body-mass-index (BMI, weight [kg] divided by height [m]^2^) and waist circumference (WC), the same level of BMI and WC has been shown to confer greater cardiovascular risk for Chinese relative to Caucasians [47,48,50,51]. This means, relatively lower levels of adiposity are sufficient to confer increased cardiovascular risk for Chinese populations. Studies have indeed shown that Asians have a higher percent body fat at lower BMI's compared to Caucasians [51-54]. As a result, many scientists conclude the that traditional World Health Organization (WHO) cutoff values for overweight (BMI >= 25) and obesity (BMI >= 30) underestimate the adverse health impact of adiposity in the Chinese population [47,48,50,51]. Thus, the obesity epidemic, and its long-term adverse health risks may be under-recognized in the modernizing People's Republic of China.

### Glycemic properties of Chinese and East Asian diet

Traditional Chinese diets are characterized and dominated by high-glycemic carbohydrates [55-57], primarily rice as the staple grain. However, Asian rice, rice porridge, and glutinous (sticky) rice are recognized to be extremely high in GI [58-61], with plain white rice having a high GI value of approximately 80 [62], which elicits postprandial glucose responses close to that of pure glucose [59]. Moreover, even higher GI values are observed for varieties of rice porridge and glutinous rice [58,63], also frequently consumed. The high GI of these foods have been strongly correlated with dyslipidemia and metabolic conditions [62,64,65]. Because intakes of rice and high GI foods are consumed in high amounts, the Chinese diet is naturally high in GL. Thus, such a diet characterized by high-glycemic starchy staple-grains would likely result in health consequences characteristic of a high GI and GL diet. Although similar higher GI diets are also common in other East Asian countries such as Japan [62], the obesity epidemic as result of rapid modernization is a much greater issue in China.

Furthermore, as China adopts lifestyle and dietary patterns of the West, consumption of added sugars, particularly in the form of sugar-sweetened beverages like soda and fruit drinks, are accompanying and compounding the traditional high-glycemic carbohydrate diet. A comparative analysis of >100 countries, including China, indicates that from 1962 to 2000, consumption of added sugars increased globally by 74 kcal/day [66]. High-fructose corn syrup, the primary sweetener found in sugared beverages, has been shown to induce rapid and dramatic spikes in blood glucose and insulin concentrations [67,68]. Consumption of such high-glycemic sugar-sweetened beverages has been consistently associated with increased systemic inflammation [69,70] and weight gain [71] and increased risk of obesity and type 2 diabetes as a result of its high-glycemic properties [34,72,73]. Moreover, this crisis is particularly troubling in China among urban children and those from high socioeconomic status (SES) backgrounds; demographic groups which have recently seen dramatic increases in fast food and soft-drink consumption [74]. Even more disconcerting, as overall wealth increases in China, Chinese children of all demographic backgrounds report a very strong desire to consume even more fast food and sugary soft-drinks if they could afford it, with as much as 72% of high SES adolescents wanting to consume such items more frequently [74].

### Compounding risks by high glycemic index and adiposity

In the past, excess risk from a high glycemic Chinese diet may not have been adverse due to high levels of physical activity and very low prevalence of overweight and obesity in the population. Unfortunately, this counterbalancing effect is disappearing in a modernizing Chinese society, particularly in urban regions, as sedentary activity and adiposity are both increasing; with both of these factors now implicated as a major contributor to excess mortality in China [2].

**Figure 1:**
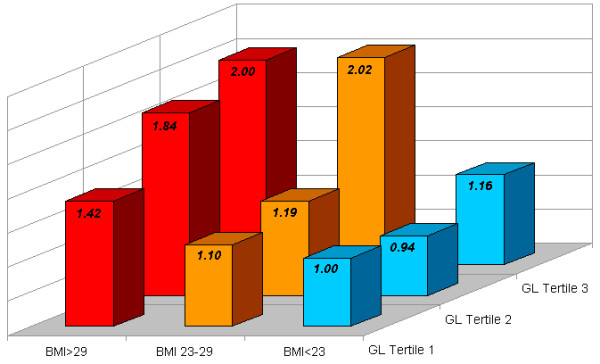
Dietary Glycemic Load and Relative Risk of Coronary Heart Disease in Women, Stratified by Body Mass Index. *P for interaction < 0.01. Adapted from updated results of Liu et al. [39]

**Figure 2:**
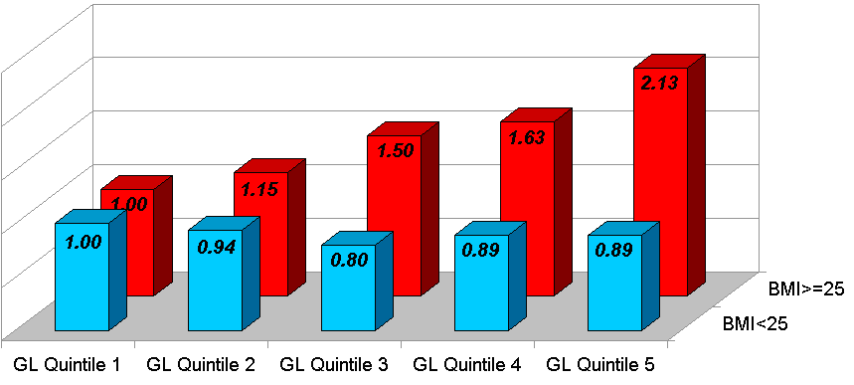
Dietary Glycemic Load and Relative Risk of Stroke in Women, Stratified by Body Mass Index. *P for interaction < 0.01. Adapted from Oh et al. [40]

**Figure 3:**
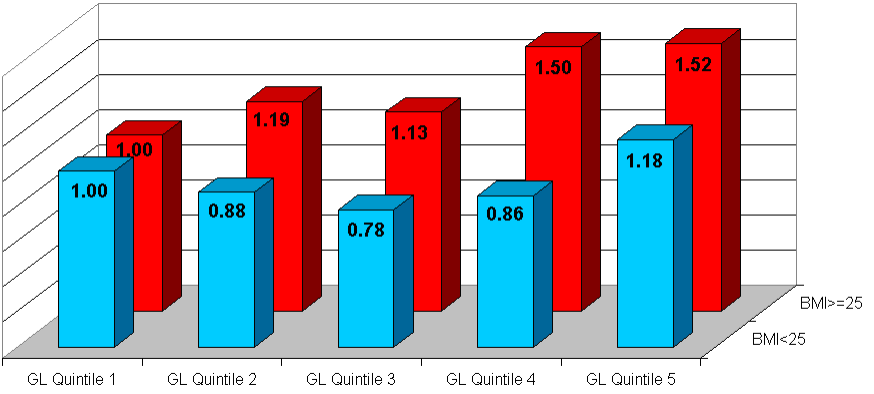
Dietary Glycemic Load and Relative Risk of Type 2 Diabetes in Chinese Women, Stratified by Body Mass Index. *P for interaction = 0.04. Adapted from Villegas et al. [35]

As previously discussed, a high GI/GL diet contributes to weight gain and obesity, as well as induces poor postprandial glucose control, and adversely increased serum lipid levels [25]. Moreover however, there exists an important biologic synergy in which a high glycemic diet elicits significantly greater adverse risks among overweight and sedentary populations. Notably, a high-glycemic diet induces a consistently significantly stronger effect on the development of type 2 diabetes [35,75], coronary heart disease [38,39], and stroke [40], especially among those with greater adiposity (BMI > 23 or BMI > 25), (all 3 diseases: P for interaction < 0.05). (See Figures 1, 2, and 3) Notably, the risk of type 2 diabetes, CHD, and stroke is not significantly elevated with increasing glycemic load among lean populations; while in contrast among overweight individuals, results show >50% increase in RR with higher intake of GL for type 2 diabetes [35], and >2-fold RR for CHD and stroke [39,40]. Moreover, similar patterns have also been repeated observed for GL and cancer risk, where high GL is more strongly associated with colorectal cancer incidence among those with higher BMI [76,77], and high-glycemic sugar-sweetened beverages more strongly linked with pancreatic cancer among those with low physical activity and/or greater adiposity [78].

Such a phenomenon of a synergistically increased adverse risk is attributed to deteriorating state of insulin-resistance and glucose control in overweight individuals [39], who are generally more susceptible and prone to uncontrolled postprandial hyperglycemia after glucose challenge from a high-glycemic meal. Additionally, clinical trial evidence also indicates that a high GI diet induces a sequence of hormonal and metabolic changes that promote excessive food intake in obese individuals [79], further compounding the vicious cycle with the effect of excess caloric intake, a risk factor for a vast majority of chronic diseases.

Thus, while a high-GI/GL diet is more adverse among overweight and obese individuals, it can be conversely expected that adhering to a low-GL diet should exert more favorable health benefits among those overweight and suffering from hyperglycemia and insulin resistance [79]. This was indeed demonstrated in a clinical trial which found significantly greater weight loss with a low-GL diet among those with underlying hyperinsulinemia and insulin resistance [37].

Currently, an estimated one-quarter of the Chinese population is overweight or obese [5], which is a likely underestimation since traditional measures of adiposity have tended to underestimate both fat mass and obesity risk in Chinese populations [47,52,54]. Most importantly, however, the recent obesity epidemic in China not only bodes ominously for increased risk of chronic disease by virtue of adiposity itself, but also predicts a rising tide of even greater adverse compounding risk from a high-glycemic diet. Furthermore, because a high-glycemic diet also promotes weight gain and energy-dysregulation in obese individuals [80], there is significant potential for the Chinese high-glycemic staple diet to also drive a vicious cycle of caloric-excess and obesity, leading to even greater risk of disease.

## Conclusion

A high glycemic index staple diet in China will become an even greater public health concern as it will compound the adverse effects of increasing adiposity, leading to dramatically increased cardiometabolic risks. Given the enormous cost to society of $18 billion/yr from diabetes and CVD morbidity in 2005, and an estimated $556 billion over the next 10 years [4], China cannot afford to ignore the astronomical impact of obesity on the health of future generations. Therefore, it is imperative that the Chinese government takes immediate action to initiate public health programs to reverse the tide of the emerging obesity epidemic in China, thereby to preemptively diffuse the enormous compounding health risks stemming from the negative convergence of obesity with a high GI Chinese diet.

## Abbreviations

GI, Glycemic index; GL, Glycemic load; CHD, Coronary heart disease; CVD, Cardiovascular disease; GDP, Gross domestic product; PRC, People's Republic of China; RR, Relative Risk; SES, Socioeconomic status

## Competing interests

The author(s) declare that they have no competing interests.

## Authors' contributions

ED and VSM contributed equally to this manuscript.

## Appendix

### Potential Policy Directions in China

The first priority is to target overweight populations with anti-obesity education and treatment options, as well as promote general education regarding lifestyle changes to prevent excess weight gain and obesity. However, general nutrition education is well-known to be less than optimal in public health effectiveness. Additionally, given the strong traditions of Chinese culture and the society's long-time reliance on rice as the staple crop – efforts to change the dietary foundation may be difficult, if not impossible. Furthermore, with the population of China exceeding 1 billion, clinical, surgical or pharmacological solutions would not likely be cost effective on such a vast population-wide scale, especially given disparities in economic resources and medical access across subpopulations and regions of China [81-84]. Thus, alternative public health policies must be developed to resolve the high-GI-obesity dilemma.

Unlike Western societies dominated by democracy as the foundation of social and policy change, China's communist government offers a unique centralized entity, which can influence society, by enforcing public nutrition policies and regulating the food supply. In an example unrelated to nutrition, the PRC government has been able to implement national programs such as the one-child-per-family law with relatively high success via a centralized structure of systematic changes in criminal law, civil law, rules of civil employment, rules of available civil services, rewards of civil obedience and disobedience, as well as other integrated societal services and governmental regulations. The PRC government also has far-reaching capabilities to directly set prices of domestic and imported goods and commodities, in addition to the international value of the currency itself.

Therefore, the Chinese government has the unique ability to implement nationwide social programs on public nutrition, as well as directly manipulate the pricing and composition of the food supply. From the aspect of social nutrition programs, it is conceivable that the PRC government could implement nationwide or region-specific public campaigns, via all forms of mass media, community-based promotion, as well as work-place promotion, on nutrition and lifestyle changes per recommended guidelines of various health organizations and reviews of chronic diseases [4,8-10,14,15] But first, social promotion must also be coupled by macro-scale changes in the food supply. Due to the already intensive nature of agriculture in China to feed its current population, it does not seem feasible to shift agriculture towards increased livestock production and protein consumption, which would require additional grain production and would further overstrain China's limited farmland. In contrast, agricultural processing of carbohydrates could potentially be shifted to production of greater proportion of lower-glycemic whole-grains rather than highly refined grains, along with changes in governmentally set prices of such commodities. However, the wider distribution of whole-grain products may be countered by decreased shelf-life and storage of such produced grains, though reconstituted whole grains would somewhat decrease rancidity. In addition, a glycemic shift in the carbohydrate composition of the food supply likely requires innovations in more efficient and improved transport and distribution of grain products.

At the same time, China should also focus on modifying the external influence of international products on traditional Chinese dietary patterns. As China continues to modernize and increase its per capita GDP, Chinese have begun to consume Western products in greater quantity such as high-glycemic sugar-sweetened beverages and fast food, which is equally or more adverse than the former with its high saturated and trans-fat content. In particular, urban Chinese children and children from high socioeconomic status (SES) backgrounds have dramatically increased fast food and soft-drink consumption [74]. Even more concerning, in light of increasing rates of household income in China, is that Chinese children of all backgrounds report strong desires to consume even more fast food and sugary soft-drinks if they could afford it, with as much as 72% of high SES adolescents wanting to consume such items more frequently [74]. Additionally, improved education of what is a healthy body weight in Chinese adolescents may be necessary, as there exists significant discordance between true healthy weight and desired body weight and obesity perception among Chinese children [85-87]; particularly worrisome are the higher-than-optimal or more overweight body sizes desired by Chinese adolescent boys [85-87] and by their parents [85]. Therefore, governmental run school-based programs, which can mandate curriculum changes to target children and adolescent weight management, may be a reasonable approach to establishing healthy weight initiatives. In addition, governmental bans, sales restrictions, and/or substantially increased tariffs on such non-domestic fast food and soft drink products may also be reasonable strategies to promote weight loss, although fear of inhibiting Western trade and financing may inhibit such governmental actions.

To date, the Chinese government has indeed begun efforts to improve the health and nutrition of its population via the development, dissemination, and implementation of a series of policies and projects [4]. One main governmental focus has been schools, where projects are achieving positive improvements in childhood obesity prevalence. An encouraging example is a multi-city school-based project, where after just 1 year, the prevalence of obesity in 8 – 14 year-olds was reduced from 21% to 14% [4].

Nevertheless, there are many potential obstacles to governmental change which should be considered. Most notably, are the initial and direct costs of various governmental programs which may be prohibitively too great. Moreover, competing economic interests from state-sponsored food industries as well as considerations of international investment in China may inhibit required changes to the food supply, food pricing, sale restrictions, and food taxation policies. Finally, and perhaps most importantly, because of the PRC government's high priority to maintain social stability – such dramatic changes to the critical and sensitive Chinese domestic food supply may indeed be difficult to elicit through the necessary governmental will to act.
